# Distinct Residential and Infiltrated Macrophage Populations and Their Phagocytic Function in Mild and Severe Neonatal Hypoxic-Ischemic Brain Damage

**DOI:** 10.3389/fncel.2020.00244

**Published:** 2020-08-10

**Authors:** Yingjun Min, Lin Yan, Qian Wang, Fang Wang, Hairong Hua, Yun Yuan, Huiyan Jin, Ming Zhang, Yaling Zhao, Jianzhong Yang, Xiangning Jiang, Yuan Yang, Fan Li

**Affiliations:** ^1^Department of Pathology and Pathophysiology, School of Basic Medical Sciences, Kunming Medical University, Kunming, China; ^2^Department of Human Anatomy and Histoembryology, School of Basic Medical Sciences, Kunming Medical University, Kunming, China; ^3^Department of Functional Experiment, School of Basic Medical Sciences, Kunming Medical University, Kunming, China; ^4^Yunnan Key Laboratory of Stem Cell and Regenerative Medicine, Institute of Molecular and Clinical Medicine, Kunming Medical University, Kunming, China; ^5^Department of Obstetrics and Gynecology, First Affiliated Hospital of Kunming Medical University, Kunming, China; ^6^Department of Psychiatry, The Second Affiliated Hospital of Kunming Medical University, Kunming, China; ^7^Department of Neurology, University of California, San Francisco, San Francisco, CA, United States; ^8^Department of Physiology, School of Basic Medical Sciences, Kunming Medical University, Kunming, China

**Keywords:** hypoxia, ischemia, microglia, monocyte-derived macrophages, phagocytosis

## Abstract

Neonatal brain injury, especially severe injury induced by hypoxia-ischemia (HI), causes mortality and long-term neurological impairments. Our previous study demonstrated activation of CD11b^+^ myeloid cells, including residential microglial cells (MGs) and infiltrating monocyte-derived macrophages (MDMs) in a murine model of hypoxic-ischemic brain damage (HIBD), with unknown functions. Here, we study the differences in the phagocytic function of MGs and MDMs to clarify their potential roles after HIBD. HI was induced in 9–10-day postnatal mice. On days 1 and 3 after injury, pathological and neurobehavioral tests were performed to categorize the brain damage as mild or severe. Flow cytometry was applied to quantify the dynamic change in the numbers of MGs and MDMs according to the relative expression level of CD45 in CD11b^+^ cells. CX_3_CR_1_^GFP^CCR_2_^RFP^ double-transformed mice were used to identify MGs and MDMs in the brain parenchyma after HIBD. Lysosome-associated membrane protein 1 (LAMP1), toll-like receptor 2 (TLR2), CD36, and transforming growth factor (TGF-β) expression levels were measured to assess the underlying function of phagocytes and neuroprotective factors in these cells. The FITC-dextran 40 phagocytosis assay was applied to examine the change in phagocytic function under oxygen-glucose deprivation (OGD) *in vitro*. We found that neonatal HI induced a different degree of brain damage: mild or severe injury. Compared with mildly injured animals, mice with severe injury had lower weight, worse neurobehavioral scores, and abnormal brain morphology. In a severely injured brain, CD11b^+^ cells remarkably increased, including an increase in the MDM population and a decrease in the MG population. Furthermore, MDM infiltration into the brain parenchyma was evident in CX_3_CR_1_^GFP^CCR_2_^RFP^ double-transformed mice. Mild and severe brain injury caused different phagocytosis-related responses and neuroprotective functions of MDMs and MGs at 1 and 3 days following HI. The phagocytic function was activated in BV2 cells but downregulated in Raw264.7 cells under OGD *in vitro*. These observations indicate that neonatal HI induced different degrees of brain injury. The proportion of infiltrated macrophage MDMs was increased and they were recruited into the injured brain parenchyma in severe brain injury. The resident macrophage MGs proportion decreased and maintained activated phagocytic function in both mild and severe brain injury, and restored neuroprotective function in severe brain injury.

## Introduction

Neonatal hypoxia-ischemia (HI) is the primary cause of mortality and long-term neurological impairments in infants. With the development of obstetric and neonatal care in recent years, neonatal mortality has been sharply reduced. However, brain injury, especially severe brain injury following neonatal HI insult, remains a clinical issue that cannot be neglected. Depending on the severity of the injury, survivors may experience different levels of post-HI impairments. Mild brain damage is associated with attention deficit-hyperactivity syndrome and minimal brain disorders, whereas severe brain damage may lead to cerebral palsy, epilepsy, cognitive dysfunction, and abnormal neurodevelopment (Okereafor et al., 2007; Ziemka-Nalecz et al., 2017; O’Driscoll et al., 2018). Due to the diverse pathological and functional outcomes of neonatal hypoxic-ischemic brain damage (HIBD), it is necessary to learn more about the underlying mechanisms for the variable severity of HI brain injuries to develop more efficient treatments.

Neuroinflammation plays a pivotal role in ischemic brain injury and recovery and is kindled by central and peripheral immune system activation, which consists of immune cell activation and cytokine release (Durafourt et al., 2012; Li et al., 2017). Cells of myeloid origin, such as microglial cells (MGs) and monocyte-derived macrophages (MDMs), are major actors in brain inflammation, a hallmark of acute brain injury. MGs, the major innate immune cells in the brain, are CD11b positive (Li et al., 2017) and coexist with another CD11b-positive cell population, MDMs, in developing brain under physiological conditions (Ginhoux et al., 2010). Increasing evidence has revealed that the activation patterns of MGs in response to HI differ throughout development (Cengiz et al., 2019). However, CD11b^+^ cells are traditionally regarded as MGs even though both MGs and MDMs express CD11b (Denker et al., 2007; Su et al., 2019; Nishihara et al., 2020). Due to a lack of specific discriminating cell markers, an open challenge is to properly characterize the roles of MGs and MDMs in neonatal brain HI injury progression and resolution. Through the use of CD45 expression levels to distinguish MDMs from MGs (Doran et al., 2019; Sun et al., 2019), the differential roles of these two populations after an injury have recently been uncovered (Martin et al., 2017). Some studies, including our previous data, have shown that MDMs enter into the brain in neonatal rat and mouse models of HI (Leonardo et al., 2008; Rocha-Ferreira and Hristova, 2015; Min and Li, 2017). However, direct evidence for MDMs invading the brain parenchyma in neonatal HI brain injury is lacking. Furthermore, the dynamic changes in the residential macrophage MG and infiltrated MDM populations and their distinct roles in establishing brain damage following neonatal HI remain unclear.

The primary functions of MGs and MDMs during central nervous system (CNS) injury are the propagation of inflammation and phagocytosis, but their neuroprotective role remains controversial. A previous study demonstrated that MGs in the postnatal day (P) 9 brain respond to HI with greater proliferation and proinflammatory cytokine release compared to MGs in the P30 brain (Ferrazzano et al., 2013). MGs play a neuroprotective role in the subventricular zone (SVZ) in neonatal HI (Fisch et al., 2020). The phagocytic activities of MGs and MDMs within the CNS are thought to play important roles in recovery, maintenance, and outcome during disease or injury (Yong and Rivest, 2009). In the developing brain, MGs are responsible for synaptic pruning and clearing of overproduced neurons and weak synapses, thereby governing brain connectivity by phagocytosis, and play a key role in organizing brain circuits during the postnatal period (Mallard et al., 2019). Therefore, MG phagocytosis in the immature brain is critical in the maintenance of behavioral function. The inflammatory function of MGs and MDMs has been shown to differ in an adult ischemia model (Zarruk et al., 2018). In this study, we investigated whether MGs and MDMs have different phagocytic functions in neonatal HI with different degrees of brain injury.

We used P 9–10 mouse pups, as this brain developmental stage is similar to newborn human infants at 37 weeks gestation (Semple et al., 2013). Mild and severe brain damage was distinguished by gross brain morphology. Changes in MG and MDM cell populations were identified by the combination of the cell markers CD11b and CD45 within 3 days after HI injury. CX3C chemokine receptor 1 (CX_3_CR_1_)^GFP^ CC motif chemokine receptor 2 (CCR_2_)^RFP^ double-transformed mice were used to identify MGs and MDMs existing in the brain parenchyma (Fumagalli et al., 2015). The expression of transforming growth factor (TGF)-β (Butovsky et al., 2014) was assessed as an indicator of the neuroprotective function of MGs and MDMs. Finally, to explore phagocytosis-related responses of MGs and MDMs after HI, we characterized the expression of toll-like receptor 2 (TLR2), lysosome-associated membrane protein 1 (LAMP1) and CD36. A FITC-dextran 40 phagocytosis assay was employed to evaluate the phagocytic function of MGs and MDMs under the oxygen-glucose deprivation (OGD) condition *in vitro*. This study provides the molecular characterization of the MGs and MDMs in the conditions of mild vs. severe brain damage, which could help us understand their distinct phenotype and roles in regulating the inflammatory responses and brain injury following neonatal HI.

## Materials and Methods

### Animals

C57BL/6J mice were purchased from Kunming Medical University. CX_3_CR_1_^GFP^ and CCR_2_^RFP^ mice were purchased from the Jackson Laboratory (Stock No. 005582 for CX_3_CR_1_^GFP^ mouse and Stock No. 017586 for CCR_2_^RFP^ mouse). The two lines were crossed to obtain CX_3_CR_1_^GFP^CCR_2_^RFP^ mice. This study was approved by the Animal Care and Use Committee of Kunming Medical University (permit# SYXK2015-0002) and was performed following the International Guiding Principles for Animals Research, as stipulated by the Council for International Organizations of Medical Sciences (1985). Mice (both C57BL/6J and CX_3_CR_1_^GFP^CCR_2_^RFP^ mice) and their offspring were group-housed with *ad libitum* access to water and food on a 12-h light/dark cycle and in a thermoregulated environment. P 9–10 mouse pups (both sexes) were used for modeling HI brain injury, and each experiment used 3–26 animals per group (the exact numbers are presented in the figures). The overall mortality was 12.5%. All efforts were made to minimize the number of mice used and their suffering.

### Mouse Model of Neonatal HI Brain Injury

P 9–10 mouse pups were used to model neonatal HI brain injury using the modified Rice-Vannucci method (Rice et al., 1981). The procedure consists of unilateral common carotid artery occlusion combined with hypoxia to produce HI injury. Briefly, P 9–10 pups of both sexes were randomly selected, placed on a surgical table maintained at 34°C–34.5°C, and anesthetized by inhalation of sevoflurane (30 μl in mixed air and oxygen). The left common carotid artery was permanently ligated. After 1.5 h of recovery and feeding with the dam, pups were placed in a hypoxia chamber (Chinese Utility Model Patent; patent number: ZL 2013-2-010770.6) filled with 8% oxygen/92% nitrogen at 34°C–34.5°C for 45 min, followed by recovery under normoxic conditions. The extent of brain injury was visually assessed by gross examination of infarct appearance. If there was no visible infarct, pups were assigned into the mild injury group (HI-M group). If there was a visible infarct, pups were assigned to the severe injury group (HI-S group). The control group (C group) consisted of littermates that did not undergo surgery or hypoxia.

### Histology Study

Mice were anesthetized with 10% chloral hydrate (0.3 ml/100 g, i.p.) at 1 or 3 days after neonatal HI and transcardially perfused with ice-cold 0.9% saline followed by 4% paraformaldehyde (unless otherwise stated, according to subsequent analysis). Then, the brains were dissected for hematoxylin-eosin (HE) staining and immunofluorescence staining.

### HE Staining

Following the behavioral testing, the animals were sacrificed for HE staining. After transcardial perfusion fixation, the brain was rapidly removed and post-fixed for 12 h at 4°C. Paraffin sections (4 μm thickness) were cut using a slicer (SLEE, CUT5062, Germany), mounted onto gelatin-coated slides, air-dried, and stored at room temperature for later use.

For HE staining, sections were dewaxed, rehydrated, and incubated with hematoxylin solution (Solarbio, Cat: H8070, China) at 22–24°C for 15 min. After washing in a hydrochloric-alcohol solution for several seconds, the sections were incubated in an eosin solution (Solarbio, Cat: G1100, China) for 1 min followed by alcohol dehydration, xylene clearing, and coverslipping.

### Immunofluorescence Staining

Studies have reported that CX_3_CR_1_ is only expressed in MGs in the brain (Hickman et al., 2019), and native brain microglia are often identified with green fluorescent protein combined with CX_3_CR_1_ (Jung et al., 2000). CCR_2_ is expressed on the peripheral blood mononuclear macrophage surface; thus, red fluorescent protein combined with CCR_2_ is often used to mark mononuclear cells (Saederup et al., 2010). Here, we used CX_3_CR_1_^GFP^CCR_2_^RFP^ double-transformed mice to distinguish MGs and MDMs in the brain parenchyma.

CX_3_CR_1_^GFP^CCR_2_^RFP^ mice were deeply anesthetized and transcardially perfused with saline followed by 4% paraformaldehyde. The brain was rapidly removed and postfixed for 24 h at 4°C. Frozen sections (20 μm thickness) were cut using a slicer (Lecai, CM1950, Germany), mounted onto gelatin-coated slides, air-dried, and stored at −20°C until use.

For immunofluorescence staining, sections were recovered at room temperature, washed with PBS, and blocked with 5% donkey serum (Jackson Immuno Research, Cat: 017-000-121, USA) for 2 h at room temperature. Then, slides were mounted with coverslips with Fluoroshield with DAPI (Sigma, F6057-20 ml, USA) and coverslipped. A fluorescence microscope (Zeiss, Axio Observer Z1, Germany) was used to visualize the slides.

### Bodyweight and Behavioral Testing

Mice were weighed at 19:00 every evening from 1 to 3 days after HI. The grip test and suspension test were performed at 1, 2, and 3 days after HI to assess the strength and fatigability of the mice. In the grip test, the front paws of all mice were placed on a wire 45 cm above the ground. Mice were released and scored according to the following system: 0 = fell off; 1 = one or both front paws gripped the wire tightly; 2 = tried to climb the wire; 3 = one or both front paws and one or both hind paws gripped the wire tightly; 4 = the front and hind paws gripped the wire and the trail wound around the wire tightly; 5 = the mouse escaped.

In the suspension test, the front paws of all mice were placed on the wire 45 cm above the ground. The time the mouse held onto the wire was scored according to the following system: 1 = 0–10 s; 2 = 11–30 s; 3 = 31 s-2 min; 4 = 3–5 min; 5 = ≥ 5 min. The experimenters who performed behavior tests were blind to the group assignment.

### Cell Separation and Flow Cytometry

For flow cytometry, single-cell suspensions were prepared from the mouse brain after perfusing with Ca^2+^/Mg^2+^-free Hanks’ balanced salt solution. The cortex was dissected on ice and enzymatically digested using a Neural Tissue Dissociation Kit (Miltenyi Biotec, Germany, cat: 130-092-628), followed by myelin removal using myelin-conjugated magnetic beads (Miltenyi Biotec, Germany, cat: 130-096-733) and an LS column (Miltenyi Biotec, Germany, cat: 130-042-401) as previously described (Li et al., 2015). The myelin-free cell fraction was centrifuged, and the cell yield was collected for flow cytometry assay. Single-cell myelin-free suspensions from different groups were centrifuged. The pellet was then resuspended in 100 μl blocking buffer containing CD16/32 (1:100, Biolegend, USA) for 10 min and incubated in fluorescence-activated cell sorter (FACS) staining buffer containing 2% fetal bovine serum (FBS, BI, Cat: 04-001-1A Israel). Then, the cells were incubated with an antibody mixture at room temperature for 30 min, washed, centrifuged, and resuspended in staining buffer. For each experiment, data from equal numbers of events (1 × 10^5^) from each group were collected on a BD C6 flow cytometer (BD Biosciences). The following combinations of antibodies with a 1:100 dilution in staining buffer were used: (1) APC anti-mouse CD45 (Biolegend)/FITC anti-mouse CD11b (Biolegend)/PE anti-mouse CD36 (Biolegend)/PerCP anti-mouse LAMP1 (Nouvs); and (2) APC anti-mouse CD45 (Biolegend)/FITC anti-mouse CD11b (Biolegend)/PE anti-mouse TLR2 (Biolegend)/PerCP anti-mouse TGF-β (Biolegend). Compensation beads (BD Bioscience, USA) were incubated with the antibody mixture (4°C, 30 min) and resuspended in staining buffer. Gating and data analysis was performed using FlowJo software (TreeStar, USA).

### Cell Culture and Oxygen-Glucose Deprivation (OGD)

The mouse MG line BV2 was kindly provided by Yuan Yun’s laboratory at Kunming Medical University. The mouse mononuclear macrophage cell line Raw264.7 was kindly provided by Bian Li’s laboratory at the First Affiliated Hospital of Kunming Medical University. Cells were plated at 1 × 10^6^ cells per 25 cm^2^ flask with high-glucose Dulbecco’s modified Eagle’s medium (DMEM; BI, Cat: 06-1055-57-1ACS, Israel) supplemented with 10% FBS (BI, Cat: 04-001-1A Israel) and 1% penicillin/streptomycin (BI, Cat: 03-031-1B, Israel). Cultures were maintained at 37°C and 5% CO_2_.

Before OGD treatment, cells were rinsed once with warm PBS, trypsinized, and plated at 2 × 10^4^ cells per 14-mm^2^ coverslip or 1 × 10^5^ cells per well in a 12-well plate. After 12 h, for OGD treatment, cells were washed with cold PBS and then refreshed with O_2_- and glucose-free DMEM (Solarbio, Cat: 90113, China). Cells were immediately placed in a sealed chamber (Billups Rothenburg, Cat: MIC-101, USA) loaded with mixed gas containing 5% CO_2_ + 95% N_2_ for 3 min at 40 L/min. The chambers were then incubated at 37°C for 4 h. Then, the cells were used in the FITC-dextran 40 phagocytosis assay.

### FITC-Dextran 40 Phagocytosis Assay

To determine the difference in the phagocytic function of BV2 and Raw264.7 cells after OGD, these two cell types were incubated with FITC-dextran 40 (TdB Consultancy AB, cat: FD40, Sweden) for 30 min at 4°C or 37°C after OGD. Following removal of FITC-dextran 40 and washing with cold PBS, the cells were coverslipped with Fluoroshield with DAPI (Sigma, F6057-20 ml, USA) and visualized with a fluorescence microscope (Zeiss, Axio Observer Z1, Germany).

For flow cytometry, the cells were trypsinized after OGD and washed twice with PBS. Gating and data analysis was performed using FlowJo software (TreeStar, USA).

### Statistical Analysis

Data analysis was performed using ImageJ (National Institutes of Health, USA), FlowJo software 7.6 (TreeStar, USA), and GraphPad Prism 6 (GraphPad Software, USA). All data are presented as the mean ± standard error of the mean (SEM), and the number of animals used (*n*) is indicated in the text and figures. Normally distributed parameters were compared using two-tailed Student’s *t*-test for two comparisons and one-way analysis of variance (ANOVA) with Bonferroni *post hoc* test for multiple comparisons. A value of *p* < 0.05 was considered statistically significant.

## Results

### Neonatal HI Induces Mild and Severe Brain Injury

To better understand the variability in neonatal HI brain injury and the underlying mechanisms, we divided HI mice into mild and severe injury groups according to the infarct appearance by gross observation 1 and 3 days after HI. The reliability of this classification was confirmed by assessing body weight, brain HE staining, and behavioral tests.

**Figure 1:**
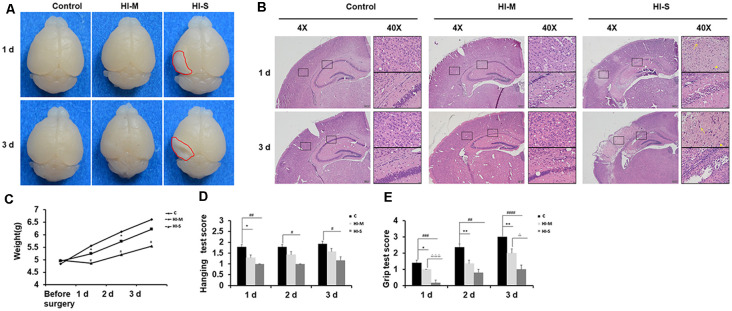
Neonatal HI induces mild and severe brain injury. **(A)** At 1 day and 3 days after hypoxia-ischemia (HI), brains with no visible infarct were classified into the mild injury group (HI-M cells are traditionally regardedgroup), and those with visible infarct (red circle) were classified in the severe injury group (HI-S group). Littermates that did not undergo HI comprised the control group (C group). **(B)** Representative images of hematoxylin-eosin (HE) staining of coronal brain sections. Normal cortex and hippocampal structure in the C group at 1 and 3 days. There were subtle cell disruption and disorientation in the cortex and hippocampus with no clear cell loss in the HI-M group. However, the HI-S group revealed signs of cell necrosis, patches of cell loss or infarct in the cortex, and throughout the pyramidal cell layer of the hippocampus at both 1 and 3 days after HI. The control C group showed normal cortical and hippocampal structure and morphology. **(C)** The weight of mice in the C group, HI-M group, and HI-S group before surgery and 1, 2, and 3 days after HI. **(D,E)** Motor performance, assessed by the hanging test **(D)** and grip test **(E)** in the C group, HI-M group, and HI-S group at 1, 2, and 3 days after HI. C, control group; HI-M, hypoxia-ischemia brain damage with mild injury; HI-S, hypoxia-ischemia brain damage with severe injury. *p < 0.05 HI-M vs. C, **p < 0.01 HI-M vs. C, ^#^*p* < 0.05 HI-S vs. C, ^##^*p* < 0.01 HI-S vs. C, ^###^*p* < 0.001 HI-S vs. C, ^####^*p* < 0.0001 HI-S vs. C, ^△^*p* < 0.05 HI-S vs. HI-M, ^△△△^*p* < 0.001 HI-S vs. HI-M, *n* = 3–26 animals per group, data are expressed as means ± standard error of the mean (SEM).

As shown in Figure 1, mice with no visible infarct on the ipsilateral side were assigned to the mild injury group (HI-M group); in contrast, mice with visible infarct appearing on the ipsilateral side of the ligated carotid artery were designated into the severe injury group (HI-S group; Figure 1A). HE staining was performed to verify the injury grade after HI in a more detailed manner. In the HI-M group, there was subtle cell disruption and disorientation in the cortex and hippocampus with no clear cell loss. However, the HI-S group revealed signs of cell necrosis, patches of cell loss or infarct in the cortex, and throughout the pyramidal cell layer of the hippocampus at both 1 and 3 days after HI (Figure 1B). The control (C) group showed normal cortical and hippocampal structure and morphology (Figure 1B). Furthermore, mice in the HI-S group had a significantly lower body weight than those in the C and HI-M groups over 3 days after the procedure (Figure 1C).

Neonatal HI mice and control mice underwent behavioral testing for 3 days. Compared to the HI-M group, the HI-S group showed more severe deficits in the grip test on day 1 and day 3 (Figure 1E). In the hanging test, although no significant differences were observed between the HI-S and HI-M groups, the HI-S group had a significantly lower score than the C group (Figure 1D).

These results suggest that neonatal HI induces different grades of brain damage and that gross examination of the ipsilateral hemisphere can be used to grade each brain as mild or severe damage. This classification was supported by HE staining, body weight, and neurobehavioral tests. Therefore, in the following experiments, we used a gross examination of the brain to distinguish mild and severe injury following HI and related the biochemical findings to the extent of severity of the injury.

### Dynamic Changes in the Residential and Infiltrated Macrophage Subpopulations in Mild and Severe Brain Injury After HI

**Figure 2:**
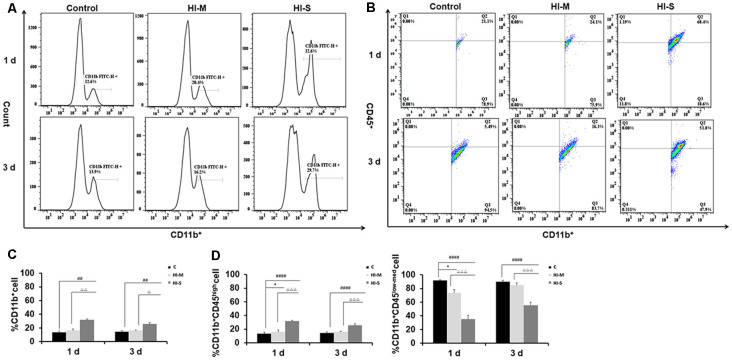
Different CD11b^+^ cell subpopulations are present in mild and severe brain injury after HI. **(A)** Representative flow cytometry histogram of CD11b^+^ fluorescence intensity at 1 day and 3 days after HI in the C group, HI-M group, and HI-S group. **(B)** The percentage of CD11b^+^CD45^low-med^ and CD11b^+^CD45^high^ fluorescence intensity at 1 day and 3 days after HI. **(C)** Flow cytometry analysis showing CD11b^+^ cells presented as a percentage of total cells at 1 and 3 days after HI. **(D)** Flow cytometry analysis showing CD11b^+^CD45^low-med^ and CD11b^+^CD45^high^ cells presented as a percentage of total CD11b^+^ cells at 1 and 3 days after HI. C, control group; HI-M, hypoxia-ischemia brain damage with mild injury; HI-S, hypoxia-ischemia brain damage with severe injury. *p < 0.05 HI-M vs. C, ^##^*p* < 0.01 HI-S vs. C, ^####^*p* < 0.0001 HI-S vs. C, ^△^*p* < 0.05 HI-S vs. HI-M, ^△△^*p* < 0.01 HI-S vs. HI-M, ^△△△^*p* < 0.001 HI-S vs. HI-M, *n* = 4–6 animals per group, data expressed as the means ± SEM.

We then asked how the macrophage subpopulations changed in response to HI in mild and severe injury. As previously described, CD45 expression can be used to distinguish residential macrophages (MGs) from infiltrated macrophages (MDMs; Prinz and Priller, 2010). Resident MGs are often defined by the occurrence of CD11b^+^CD45^low-med^ cells, whereas the cells identified as CD11b^+^CD45^high^ represent infiltrated MDMs (Maria and Watters, 2012; Bedi et al., 2013; Denieffe et al., 2013; Sushanta et al., 2017). We employed flow cytometry analysis to assess the total number of CD11b^+^ cells and identify specific phenotypes within this population after HI (Figures 2A–D).

As shown in Figure 2, the HI-S group had significantly more CD11b^+^ cells than the C group and HI-M group at both 1 and 3 days after HI insult, whereas there were no differences in CD11b^+^ cell number between the HI-M group and C group (Figures 2A,C). Then, we further distinguished the subpopulation of CD11b^+^/CD45^+^, MGs (CD11b^+^CD45^low-med^ cells) and MDMs (CD11b^+^CD45^high^ cells). In the HI-S group, the percentage of MGs was significantly lower and that of MDMs was significantly higher than those in the C group and HI-M group at both 1 and 3 days after HI (Figures 2B,D). Interestingly, despite the lack of an overall increase in CD11b^+^ cells in the HI-M group, the percentage of MGs was lower and that of MDMs was higher than those in the C group at 1 day post-HI (Figures 2B,D).

These data suggest that resident MG (CD11b^+^CD45^low-med^ cells) proliferation is limited in neonatal HI, while MDMs (CD11b^+^CD45^high^ cells) are recruited toward the injury cortices, resulting in a sharp increase in the proportion of MDMs among all CD11b^+^ cells in both HI-M and HI-S groups after HI. These changes sustained longer in the HI-S brains.

### MDMs Invade the Brain Parenchyma in Severe Brain Injury After HI

**Figure 3:**
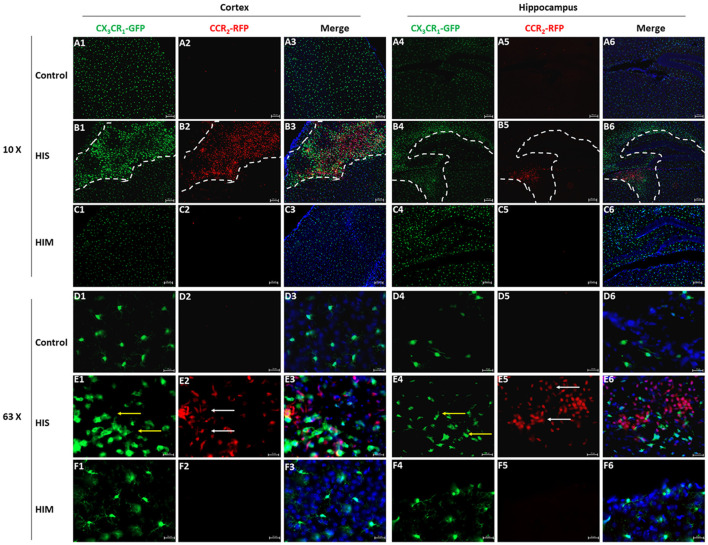
Monocyte-derived macrophages (MDMs) invade the brain parenchyma in severe brain injury after HI. **(A–F)** Representative photomicrographs of the cortex **(A1–F3)** and hippocampus **(A4–F6)** from CX_3_CR_1_^GFP^CCR_2_^RFP^ mice in the HI-S group **(B1–B6,E1–E6)** and HI-M group **(C1–C6,F1–F6)** at 3 days after neonatal HI in the ipsilateral **(B1–B6,E1–E6)** and contralateral **(A1–A6,D1–D6)** brain. White arrow demonstrates CCR_2_^RFP^-positive cells, and yellow arrow demonstrates CX_3_CR_1_^GFP^-positive cells. C, control group; HI-M, hypoxia-ischemia brain damage with mild injury; HI-S, hypoxia-ischemia brain damage with severe injury. *n* = 3 animals per group.

Although we detected the existence of MDMs in the ipsilateral hemisphere, flow cytometry cannot exclude the possibility of the existence of these cells in brain blood vessels. To confirm the presence and changes in MG and MDM populations in the parenchyma, CX_3_CR_1_^GFP^CCR_2_^RFP^ double-transformed mice were used to identify MGs and MDMs *in viv*o (Jung et al., 2000; Saederup et al., 2010). CCR_2_^RFP^-labeled peripheral mononuclear cells were detected in the ipsilateral cortex and hippocampus of the HI-S group at 3 days after HIBD (Figure 3). At this same time point, a group of round CCR_2_^RFP^-labeled MDMs (Figure 3, white arrows) was observed at the bottom of the third ventricle. In addition to a group of round ameboid CX_3_CR_1_^GFP^-labeled MGs, many CCR_2_^RFP^-labeled MDMs also appeared in the necrotic area of the brain cortex, some of which were round and some were of a long spindle shape. CX_3_CR_1_^GFP^-labeled microglia (Figure 3, red arrows) also exhibited two very different morphologies in the ipsilateral cortex and hippocampus: one is typical for the quiescent cells with a small cell body and branches of many different lengths, and the other representing activated cells with a larger cell body, shorter branches, and a round ameboid shape. However, the presence of CCR2^RFP^ cells was not observed in the HI-M group (Figures 3A1–F6), indicating that peripheral mononuclear cells invaded the brain parenchyma after HIBD in the cases of severe injury. To further assess the function of resident MGs and MDMs post-HI, we assessed the expression of several functional proteins to examine the neuroprotective and phagocytic functions of the MGs and MDMs.

### The Expression of TGF-β in CD11b^+^ Cells in Mild and Severe Brain Injury After HI

**Figure 4:**
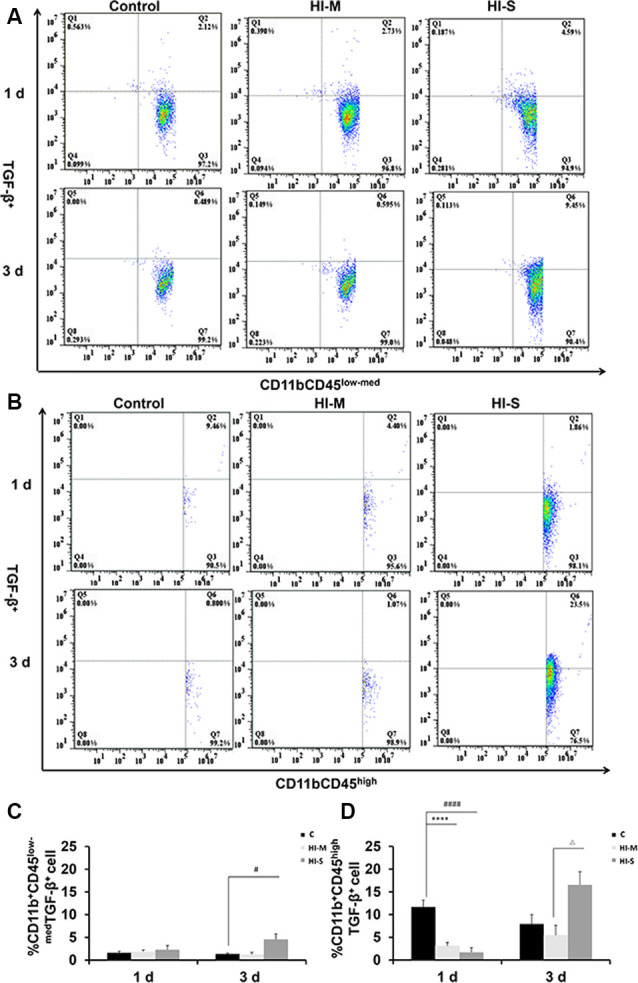
Expression of the transforming growth factor (TGF-β) on CD11b^+^CD45^low-med^ and CD11b^+^ CD45^high^ cells in mild and severe brain injury after HI. **(A)** The percentage of TGF-β^+^/CD11b^+^CD45^low-med^ fluorescence intensity at 1 day and 3 days after HI in the C group, HI-M group, and HI-S group. **(B)** The percentage of TGF-β^+^/CD11b^+^CD45^high^ fluorescence intensity at 1 day and 3 days after HI. **(C)** Quantification of TGF-β^+^/CD11b^+^CD45^low-med^ cells at 1 and 3 days after HI. **(D)** Quantification of TGF-β^+^/CD11b^+^CD45^high^ cells at 1 and 3 days after HI. C, control group; HI-M, hypoxia-ischemia brain damage with mild injury; HI-S, hypoxia-ischemia brain damage with severe injury. ****p < 0.0001 HI-M vs. C, ^#^*p* < 0.05 HI-S vs. C, ^####^*p* < 0.0001 HI-S vs. C, ^△^*p* < 0.05 HI-S vs. HI-M, *n* = 4–6 animals per group, data expressed as the means ± SEM.

To further assess the function of resident MGs and MDMs post HI, we used TGF-β as an indicator to determine the neuroprotective function of MGs and MDMs by flow cytometry. As shown in Figure 4, TGF-β expression in CD11b^+^CD45^high^ cells at 1 day post-HI was significantly lower in both the HI-M and HI-S groups than in the C group (Figures 4B,D). However, at 3 days following HI, TGF-β expression in CD11b^+^/CD45^low-med^ cells was significantly higher in the HI-S group than in the C group (Figures 4A,C). These data showed that MGs in the HI-S group produce TGF-β at 3 days following injury, whereas TGF-β expression in MDMs was inhibited in both the HI-M and HI-S groups at 1 day after HI.

### The Expression of TLR2 and LAMP1 on CD11b^+^ Cells in Mild and Severe Brain Injury After HI

To further assess the function of resident MGs and MDMs post-HI, the expression of some phagocytosis-related markers, TLR2 and LAMP1, on MGs and MDMs were studied by flow cytometry. TLR2 is considered a direct indicator of phagocytosis, while LAMP1 is a lysosomal marker used in autophagy assessments (Reinehr et al., 2020).

**Figure 5:**
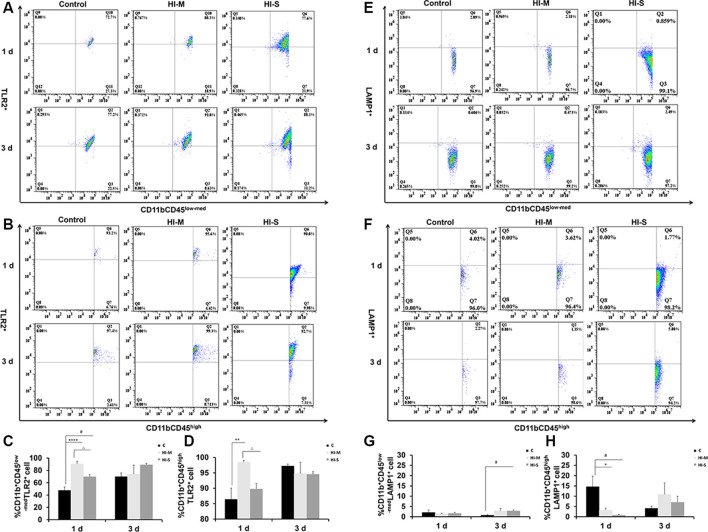
The expression of toll-like receptor 2 (TLR2) and lysosome-associated membrane protein 1 (LAMP1) on CD11b^+^CD45^low-med^ and CD11b^+^ CD45^high^ cells in mild and severe brain injury after HI. **(A)** Percentage of TLR2^+^/CD11b^+^CD45^low-med^ fluorescence intensity at 1 day and 3 days after HI in the C group, HI-M group, and HI-S group. **(B)** The percentage of TLR2^+^/CD11b^+^CD45^high^ fluorescence intensity at 1 day and 3 days after HI. **(C)** Quantification of TLR2^+^/CD11b^+^CD45^low-med^ cells at 1 and 3 days after HI. **(D)** Quantification of TLR2^+^/CD11b^+^CD45^high^ cells at 1 and 3 days after HI. **(E)** The percentage of LAMP1^+^/CD11b^+^CD45^low-med^ fluorescence intensity at 1 day and 3 days after HI in the C group, HI-M group, and HI-S group. **(F)** The percentage of LAMP1^+^/CD11b^+^CD45^high^ fluorescence intensity at 1 day and 3 days after HI. **(G)** Quantification of LAMP1^+^/CD11b^+^CD45^low-med^ cells at 1 and 3 days after HI. **(H)** Quantification of LAMP1^+^/CD11b^+^CD45^high^ cells at 1 and 3 days after HI. C, control group; HI-M, hypoxia-ischemia brain damage with mild injury; HI-S, hypoxia-ischemia brain damage with severe injury. *p < 0.05 HI-M vs. C, **p < 0.01 HI-M vs. C, ^#^*p* < 0.05 HI-S vs. C, ^△^*p* < 0.05 HI-S vs. HI-M, ****p < 0.0001 HI-M vs. C, ^#^*p* < 0.05 HI-S vs. C, ^△^*p* < 0.05 HI-S vs. HI-M, *n* = 4–6 animals per group, data expressed as the means ± SEM.

At 1 day post HI, the HI-M group had lower levels of LAMP1^+^ MDMs than the C group but no significant differences in LAMP1^+^ MGs (Figures 5E–H). In contrast, the HI-M group had significantly higher levels of TLR2^+^ MGs and TLR2^+^ MDMs than the C group (Figures 5A–D). These changes were not observed at 3 days post HI.

Similarly, the HI-S group had lower levels of LAMP1^+^ MDMs and higher levels of TLR2^+^ MGs than the C group at 1 day after HI (Figures 5A–D) but no significant differences in LAMP1^+^ MGs (Figures 5E–H). At 3 days following HI, LAMP1^+^ MGs were significantly upregulated in the HI-S group (Figures 5E–H). These data suggest that MGs (CD11b^+^CD45^low-med^) and MDMs (CD11b^+^CD45^high^) showed different phagocytosis-related responses in both mild and severe brain injury in the days immediately following neonatal HI.

### The Expression of CD36 in CD11b^+^ Cells in Mild and Severe Brain Injury After HI

**Figure 6:**
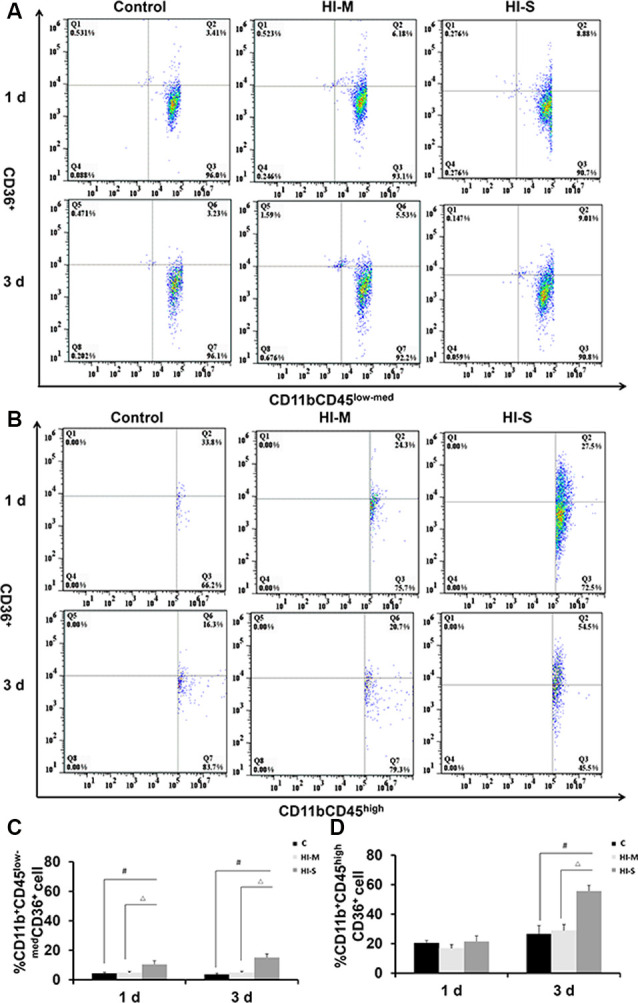
The expression of CD36 on CD11b^+^CD45^low-med^ and CD11b^+^CD45^high^ cells in mild and severe brain injury after HI. **(A)** The percentage of CD36^+^/CD11b^+^CD45^low-med^ fluorescence intensity at 1 day and 3 days after HI in the C group, HI-M group, and HI-S group. **(B)** The percentage of CD36^+^/CD11b^+^CD45^high^ fluorescence intensity at 1 day and 3 days after HI. **(C)** Quantification of CD36^+^/CD11b^+^CD45^low-med^ cells at 1 and 3 days after HI. **(D)** Quantification of CD36^+^/CD11b^+^CD45^high^ cells at 1 and 3 days after HI. C, control group; HI-M, hypoxia-ischemia brain damage with mild injury; HI-S, hypoxia-ischemia brain damage with severe injury. ^#^*p* < 0.05 HI-S vs. C, ^△^*p* < 0.05 HI-S vs. HI-M, *n* = 4–6 animals per group, data expressed as the means ± SEM.

A previous study reported that CD36 can negatively regulate TLR2 expression following adult stroke (Li et al., 2015). In this study, we examined the potential modulatory mechanisms of TLR2 related to CD36. As shown in Figure 6, the number of CD36^+^ cells among MGs in the HI-S group was substantially higher than that in the C group at 1 and 3 days after HI, while no differences were observed between the HI-M group and C group (Figures 6A–D). The number of CD36^+^ cells among MDMs in the HI-S group was higher than that in the C group at 3 days (Figures 6A–D). Combined with the TLR2 results, our data indicate that CD36 may not completely regulate the phagocytic ability of CD11b^+^ cells in our neonatal HI model.

### The Phagocytosis Ability of MGs and MDMs *in vitro*

**Figure 7:**
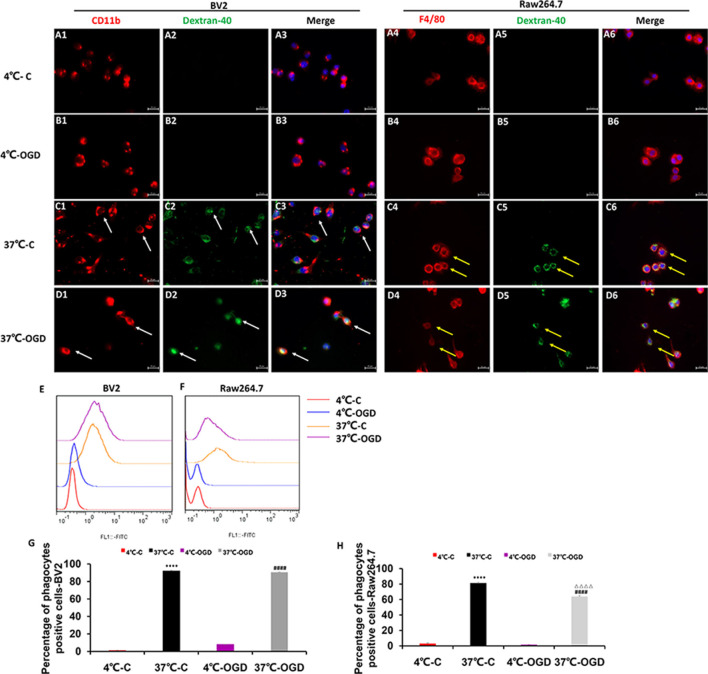
The phagocytic ability of MG and MDM cells *in vitro*. **(A–D)** Representative images showing the change in phagocytosis in the mouse MG line BV2 **(A1–D3)** and the mouse mononuclear macrophage cell line Raw264.7 **(A4–D6)** after oxygen-glucose deprivation (OGD), confirming that the cells engulfed fluorescently labeled dextran-40. **(E,F)** Representative flow cytometry images showing the change in phagocytosis in BV2 **(E)** and Raw264.7** (F)** cells after OGD. **(G)** Quantification of the percentage of phagocytic cells after OGD in the BV2 cell line. **(H)** Quantification of the percentage of phagocytic cells after OGD in the Raw264.7 cell line. C, control group; OGD, oxygen and glucose deprivation group, ****p < 0.001 37°C-C vs. 4°C-C, ^####^*p* < 0.0001 37°C-OGD vs. 4°C-OGD, ^△△△△^*p* < 0.001 37°C-OGD vs. 37°C-C.

We performed *in* *vitro* immunofluorescence and flow cytometry analyses on a mouse MG line (BV2) and a mouse mononuclear macrophage cell line (Raw264.7) to identify differences in the phagocytic abilities of these cells under OGD condition. We observed green fluorescence-labeled dextran-40 in both mononuclear macrophages (Figures 7A1–D6) and MGs (Figures 7A1–D6) under control and OGD conditions, suggesting that both mononuclear macrophages and microglia were able to engulf dextran-40. Quantitative analysis of green dextran-40 in each cell by flow cytometry revealed that after OGD, the phagocytic function of microglia was higher than that under normal condition, while that of mononuclear macrophages was lower (Figures 7E–H). Consistent with our findings *in* *vivo*, these results suggest that the phagocytosis of mononuclear macrophages was inhibited after OGD.

## Discussion

We demonstrated that neonatal brain HI resulted in a rapid decrease in the proportion of residential macrophage MGs and a sharp increase in the proportion of infiltrated MDMs in the brain parenchyma of the infarct region. At 3 days after injury, MGs rather than MDMs comprised the major macrophage population and might play a neuroprotective role, although the proportion of MGs and MDMs in CD11b^+^ cells was no significant difference at 1 day after HI injury. Different MGs and MDMs responses were observed in mild vs. severe brain injury.

The Vannucci neonatal HI model is known for its intrinsic variability. As expected, the degree of brain injury was found to be variable in our model (Wu et al., 2017; Muzzi et al., 2018). Since biochemical changes and cellular responses are related to the extent of the injury, we separated the mice into mildly and severely injured groups by gross examination of brain infarct, which is consistent with histology and associated neurobehavioral evaluations. The HI-injured mice gained significantly less weight compared to the control pups in the first 3 days indicating an impaired ability to feed. The resultant malnutrition may modify the evolution of brain inflammation and injury that needs further investigation. Using this approach, we characterized the molecular and morphological changes of residential macrophage MGs and infiltrated MDMs, as they are the major players in neuroinflammation, which is one of the pivotal mechanisms in neonatal brain HI injury.

MG and MDM subpopulations were first identified by CD11b and CD45 expression. CD11b is one of the most commonly used surface markers to investigate macrophages under physiological and pathophysiological conditions. CD45 is a transmembrane glycoprotein expressed by cells of hematopoietic origin. MGs express low constitutive levels of CD45, and even after activation, they still maintain a lower level of CD45 than MDMs (Fumagalli et al., 2015). Thus, we used CD11b^+^/CD45^low-med^ and CD11b^+^/CD45^high^ labeling to distinguish resident macrophage MGs and MDMs, respectively, by flow cytometry (Denieffe et al., 2013; Trahanas et al., 2015). Consistent with previous studies (Yao et al., 2013), an increase in CD11b^+^ cells was detected in our severe brain HI injury model. Unexpectedly, compared with the control animals, the mice with both mild and severe HI brain injury exhibited a lower proportion of MGs among total CD11b^+^ cells but a higher proportion of MDMs. In the animals with mild injury, this change was only seen at 1 day after injury, whereas it was observed at both 1 and 3 days after injury in cases of severe injury. These results are in line with a recent study showing that MGs are downregulated in the SVZ in a neonatal brain HI model (Fisch et al., 2020). However, our results are not consistent with other studies demonstrating that MGs are the major population after an adult or neonatal brain injury (Denker et al., 2007; Plemel et al., 2020), despite their inhibitory function in monocyte infiltration (Plemel et al., 2020). Our findings using the CX_3_CR_1_^GFP^CCR_2_^RFP^ double-transformed mice suggest that MDMs invade the brain parenchyma, and their recruitment is a sensitive and early incident following neonatal HI. Interestingly, rounded and elongated spindle-like CCR_2_^RFP^-labeled MDMs were observed in the necrotic area of the brain cortex, but only rounded MDMs existed in the bottom of the third ventricle. Morphology is highly related to the function and origin of MDMs, but the significance of the diverse morphologies of MDMs requires further study. Transmigration of leukocytes requires coordinated activation of leukocytes and endothelium. The presence of CD11b on peripheral leukocytes is required in the early transmigration process for “tethering” and subsequent rolling along the endothelium. A significant increase in CD11b expression on peripheral leukocytes after neonatal ischemia-reperfusion has been reported (Denker et al., 2007). In our model, enhanced CD11b expression on MDMs was also detected at 1 day after injury. Cell proliferation and migration require energy consumption, and MGs consume energy in an ATP-dependent manner (Atkinson et al., 2004; Masuda et al., 2011; Gimeno-Bayãn et al., 2014). Thus, MGs are highly susceptible to energy deficits. Unlike MGs, MDMs can switch their metabolism to anaerobiosis and remain viable in hypoxic/ischemic conditions by regulating HIF-1α and nuclear factor κB transcription factor families (Taylor and Cummins, 2009; Sica et al., 2011; Semenza, 2012; Riboldi et al., 2013). After HI, ATP concentrations in the brain decrease, and MG ATP production is lower, resulting in limited MG proliferation. However, MDMs can acquire ATP by anaerobiosis and thus be able to enter the brain. Our data showed that MDMs proportion increased in CD11b positive macrophages in the brain after neonatal brain HI injury. MDMs and MGs have distinct functions and possibly mutual regulation mechanisms that warrant further investigation.

Despite the traditional view that MGs are biphasic in brain injury (Ma et al., 2017), we found that MGs rather than MDMs produced TGF-β, which is considered as an essential neuroprotective factor of MGs in some pathophysiological conditions (Dobolyi et al., 2012; Bialas and Stevens, 2013; Butovsky et al., 2014). Of note, TGF-β expression in MDMs was inhibited at 1 day in both the HI-M and HI-S groups. In a previous study, researchers used the CSF1R/c-Kit inhibitor PLX3397 to effectively and specifically deplete MGs in mice without inducing apparent abnormalities in neurological function. PLX3397 treatment had no prominent impact on peripheral myeloid cells. Furthermore, this study demonstrated that MGs are beneficial to the adult ischemic brain through initial astrocyte neuroprotective functions (Jin et al., 2017). MGs can also provide trophic support to neurons and endothelial cells, notably *via* their production of brain-derived neurotrophic factor (BDNF), insulin-like growth factor (IGF)-1/2, and TGF-β. Disrupted growth factor production in MGs interrupts cortical layer formation in physiological conditions (Cho et al., 2013; Ueno et al., 2013). Therefore, we suggest that MGs rather than MDMs provide neuroprotection through TGF-β in severely injured neonatal HI brains.

Phagocytosis is particularly important for neonatal brains due to its role in synaptic pruning, as well as in cleaning the debris resulting from apoptotic neuronal death, which is remarkably higher in the neonatal brain than in the adult brain post-ischemia (Hu et al., 2000). We found that the expression of TLR2, a key marker of phagocytosis (Kochan et al., 2012), was elevated in MGs but not in MDMs in both mildly and severely injured brains at 1 day after HI. The phagocytic activity can also be labeled by LAMP1, an activation marker of lysosomes (Perego et al., 2011). A decrease in LAMP1 expression was observed in MDMs at 1 day post HI, suggesting that MGs are the main cells that perform phagocytosis at 1 day after HI injury. However, at 3 days after HI, the proportion of LAMP1^+^ MGs in the HI-S group was higher than that in the C group. These data indicate that the lysosomal function of MGs is activated at 3 days after severe HI injury and that MGs and MDMs exhibit sustained activation. We previously reported that CD36 regulates TLR2 expression in MGs. In the absence of CD36, TLR2 expression in MGs is increased, and brain injury is aggravated (Li et al., 2015). However, inconsistent with this finding, we found increased expression of TLR2 in MGs at 1 day after HI, while CD36 expression also remained active at 1–3 days after HI injury in the HI-S group. This discrepancy could be explained by the differences in the model used. In the previous study, we used a neonatal model of transit focal cerebral ischemia-reperfusion, while the global hypoxia-ischemia model was used here. Possibly, the surface expression of TLR2 on MGs is not solely regulated by CD36, and other mechanisms are involved as well. We also found increased TLR2 expression in MDMs in the HI-M group at 1 day after HI and increased CD36 expression in the HI-S group at 3 days after HI. Together, these observations suggest that MGs have more efficient phagocytic activity than MDMs and that TLR2 expression in MGs and MDMs is not completely controlled by CD36 in neonatal HI.

The cell culture data provided more direct evidence for the different phagocytic function of MDMs and MGs after OGD, an *in vitro* model of HI. BV2 MGs exhibited a phagocytic reaction after OGD, whereas the phagocytic reaction in monocytes was inhibited under the same condition. Our *in* *vitro* data and *in vivo* results both suggest that MGs have a phagocytic function within 3 days after neonatal brain HI injury. However, a recent study found that MDMs suppress MG phagocytic activity, whereas MGs enhance phagocytosis by MDMs (Greenhalgh and David, 2014). Blocking the infiltration of MDMs into the CNS alters MG inflammatory gene expression, increases chronic MG activation, and impairs functional recovery after adult spinal cord injury (Greenhalgh et al., 2018). The interactions between these two cell types that modulate their specific function in HIBD require further study.

## Conclusion

Activation of distinct inflammatory cells and associated pathways as a consequence of brain insult may affect the course of injury in different and even opposing ways. We found that the number of CD11b^+^ cells in severely injured brains sharply increased, mainly due to an increase in infiltrated MDMs, which were recruited into the injured brain parenchyma. Although MGs and MDMs were active in the same “theater,” they exhibited different activation patterns. Phagocytosis activation in MGs and inhibition in MDMs at 1 day after injury was detected in both mild and severe brain injury, and thus, the phagocytic activity of MGs and MDMs may be defined as a sensitive indicator of brain injury. Although the proportion of resident macrophage MGs was lower than control after brain injury, they remained active phagocytic function through enhanced TLR2 expression in both mild and severe brain injury. MGs also restored neuroprotective function possibly through TGF-β in severely injured brains.

## Data Availability Statement

All datasets presented in this study are included in the article.

## Ethics Statement

The animal study was reviewed and approved by the Animal Care and Use Committee of Kunming Medical University (permit# SYXK2015-0002).

## Author Contributions

YM and LY performed the statistical analysis. LY and QW collected weight and behavioral measurements. YM and HH performed cell culture and FITC-dextran 40 phagocytosis assays. LY, FW, and YYuan performed immunofluorescence staining. LY, JY, and HJ performed HE staining. YM, MZ, and YZ prepared the figures. FL designed the experiments. FL, YYang, and XJ wrote and revised the manuscript. All authors contributed to the article and approved the submitted version.

## Conflict of Interest

The authors declare that the research was conducted in the absence of any commercial or financial relationships that could be construed as a potential conflict of interest.
